# Carbon effect calculation and upgrading strategy of agricultural land consolidation project in urban edge of Three Gorges Reservoir Area

**DOI:** 10.3389/fchem.2022.1022644

**Published:** 2022-09-29

**Authors:** Wei Yang, Xiaohua Li, Weihua Li, Yutao Zhang, Haizhen Zhang, Yuhe Ran

**Affiliations:** ^1^ School of Resource and Environmental Engineering, Anshun University, Anshun, Guizhou, China; ^2^ College Rural Revitalization Research Center of Guizhou, Anshun University, Anshun, Guizhou, China; ^3^ School of Chemistry and Chemical Engineering, Anshun University, Anshun, China; ^4^ School of Continuing Education, Chongqing Vocational Institute of Engineering, Chongqing, China; ^5^ Administrative Committee of Anshun High tech Industrial Development Zone, Anshun, Guizhou, China

**Keywords:** agricultural land consolidation, ecosystem biomass, biomass carbon, carbon effect accounting, Shiyan town

## Abstract

Under the background of promoting the construction of ecological civilization and the goal of carbon peak and carbon neutralization, it is of great significance to explore the measurement method and improvement strategy of the carbon effect of agricultural land consolidation. Based on a quantitative analysis and the whole life cycle of land consolidation, this study constructed a carbon effect accounting and analysis framework of agricultural land consolidation project from three stages of project initiation and design, project implementation, and operation management. Taking the agricultural land consolidation project in the Shiyan town on the urban edge of the Three Gorges Reservoir Area as a case, this study made empirical analysis and calculation and analyzed the carbon effect and influencing factors in different consolidation stages. The results showed that the overall carbon effect in the project area was a carbon source. The net carbon emission generated by the project construction was 8358t, which was mainly from workers input and concrete carbon emission; the carbon storage brought about by the adjustment of land use structure was 2,378.20t, which mainly came from the carbon storage increment of newly cultivated land; the carbon storage generated by the agricultural ecosystem was 1,100.04t, which was mainly based on the increase of cultivated land and the improvement of cultivated land quality; the carbon emission from agricultural production activities was 18.18t. Research conclusions: ① the carbon source effect of engineering construction is obvious. Artificial input and concrete are the main carbon sources in the hilly area at the edge of the metropolis; ② the adjustment of land use structure is manifested as a carbon sink effect, which mainly comes from the contribution of carbon storage of newly increased cultivated land; ③ the carbon effect of project operation and management may be a carbon source in the short term, and the long-term effect should be exerted; and ④ based on the concept of whole life cycle, promoting ecological land consolidation, optimizing project design, reasonably arranging consolidation projects, and strengthening operation management are effective measures to improve the carbon effect of land consolidation projects, which are conducive to the realization of the “double carbon” goal.

## 1 Introduction

Global warming has led to a series of ecological and environmental problems, which have brought severe challenges to human survival and development. Greenhouse gas emission reduction has become a global issue (Dong et al., 2008). Under the goal of carbon peak and carbon neutrality, the implementation of carbon reduction is an important strategy for China’s economy and society to achieve green and low-carbon development. The assessment report of the Intergovernmental Panel on Climate Change (IPCC) pointed out that the main cause of global warming was the greenhouse gas emissions from burning fossil fuels and land use by human activities (IPCC, 2001). According to the data released by the World Resources Research Institute and Climate Watch in 2016, 73.2% of global greenhouse gases came from energy consumption and 18.4% from agriculture, forestry, and land use (Wu et al., 2021). Some scholars estimated that the carbon emissions from land use and change, from 1850 to 1998, accounted for 1/3 of the total carbon emissions from human activities in the same period (Watson et al., 2000; Houghton et al., 2012); from 1850 to 2000, the net emission of CO_2_ to the atmosphere due to land use change reached 156pg, of which 87% came from deforestation (Houghton et al., 1999; Watson et al., 2000). The impact of land use/land cover change (LUCC) on the carbon balance of terrestrial ecosystems has become the focus of research on global change and terrestrial carbon cycle (Gregorich et al., 1998). The carbon effect of land use and emission reduction measures are important ways to deal with global climate warming, which are of great concern to governments and scholars (Zhang et al., 2018).

Agricultural land consolidation is the reorganization of agricultural land use structure and ecosystem (Gao, 2003). It is an important way to coordinate the relationship between people and land, and one of the largest human activities to change the land use pattern and affect the terrestrial ecosystem in China (Zhang T et al., 2014; Wang et al., 2018), which directly affects the carbon cycle of the ecosystem. On the one hand, the strong disturbance of soil and the destruction of biomass caused by the project construction will directly affect the ecosystem in the project area. Meanwhile, the input of materials such as cement, steel and the consumption of energy such as gasoline and diesel will affect the carbon pool balance of the regional ecosystem.

China’s rural land consolidation is developing rapidly and on a large scale, which has a prominent impact on the rural ecological environment, ecosystem biomass, and carbon emissions. According to the National Land Consolidation Plan (2016–2020), during the 13th Five-Year Plan period, 1.3333 million hm^2^ of cultivated land will be replenished, 13.3333 million hm^2^ of low and medium cultivated land will be transformed, and 400,000 hm^2^ of rural construction land will be consolidated (The Ministry of Land and Resources of PRC The National Development and Reform Commission, 2017). Under the background of ecological civilization construction, the country has made great investment and continued promotion, which has made rural land consolidation a focus in the field of land resources management, and will continue to affect rural economic and social development and ecological environment construction.

At present, the planning, design, and construction practice of the agricultural land consolidation project pay more attention to the regional landscape pattern, soil erosion, and environmental pollution and pay less attention to the carbon effect of project implementation.

Domestic and foreign scholars have studied the carbon effect of agricultural land consolidation; one is to directly study the relationship between land use and carbon emissions, and the other is to indirectly study the carbon emissions caused by land use changes (Han et al., 2016), focusing on soil carbon content changes, energy, and material consumption, ecological compensation policies, and other contents (Tan et al., 2011; Guo et al., 2016; Zhang et al., 2016). Some scholars have also analyzed land consolidation types from the perspective of geomorphology types (Jin et al., 2013). In general, most of the existing studies are qualitative discussions and few quantitative calculations. The research on carbon emissions in different implementation stages of land consolidation projects and after the implementation of land consolidation projects is not enough. In addition, from the research area, there are few studies on the carbon effect of land consolidation in the Three Gorges Reservoir area, especially in the urban fringe. Therefore, this study reasonably absorbs the existing research results, takes the rural land consolidation project of the Shiyan town on the edge of the metropolitan area of the Three Gorges Reservoir Area in Chongqing as a case, adopts the quantitative method, and calculates the carbon effect and influencing factors at different stages based on the life cycle of agricultural land consolidation, so as to provide reference for the ecological transformation and development of agricultural land consolidation in the metropolitan area edge. It provides a basis for the formulation of measures to reduce carbon emission and improve carbon effect in land consolidation regions, helps achieve the goal of carbon peak and carbon neutrality, and further enriches the theory and methods of low carbon and ecological land consolidation.

## 2 Overview of study area

The project area is located in Shiyan Town, Changshou District, Chongqing City, between 107°10′45″–107°14′08″E and 30°03′35″– 30°05′45″N, involving three villages including Muer, Zaojue, and Jianxin. The project area is located in the south of the parallel ridge Valley Liangping Syncline in the east of Sichuan Basin, mainly developed in the middle hilly terrain, and the micro landform is shallow hilly zone dam landform, with a relative height difference of 50–160 m and an average elevation of 370m; Longxi River in the area, with an average annual flow of 54 m^3^/S; The groundwater has pressure bearing capacity and modulus is 27,000 m^3^/km^2^. It belongs to subtropical humid monsoon climate. The annual average temperature is 17.45°C, annual accumulated temperature of ≥0 °C is 6,423.7°C, annual accumulated temperature of ≥10°C is 5,783.75°C, frost free period is 331 days, average annual sunshine duration is 1,245.1 h, and average rainfall is 1117 mm, which is conducive to the growth of a variety of crops; the vegetation is mainly subtropical evergreen broad-leaved forest, with 184 tree species and 56 cultivated plants. The grain crops are mainly rice, corn, wheat, and sweet potato, and cash crops are mainly rapeseed and vegetables. The soil is brown purple soil and neutral purple mud paddy soil, with pH value of slightly acidic to slightly alkaline, and the thickness of soil layer ≥40 cm. Corn, sweet potato, and wheat can be planted in dry land three times a year; single cropping rice in the dam paddy field is mainly planted; and rice and wheat or rice and rapeseed planted in paddy fields in slope valley are mainly double cropping. The soil has high natural fertility, wide suitability for cultivation, and wide suitability for planting. The total area is 553.83 h m^2^, population is 5,443, agricultural land is528.14 h m^2^, unused land is 25.69 h m^2^, and land reclamation rate is 64.63%. Land consolidation projects include land leveling, irrigation, drainage, field roads, farmland shelter forests, and other projects. The artificial leveling earthwork is 416,500 m^3^, artificial tamping Earth barrier is 123,302 m^3^, dry block stone sill 10090 m^3^, new field road is 5 km, maintenance field road is 10.34 km, roads for production is 32.14 km, drainage and irrigation ditch are 28.083 km, new irrigation channel is 28.36 km, impounding reservoir is 67, desilting basin is 32,970, and shelter forest belt is 111.21 km.

## 3 Data sources and processing

### 3.1 Data sources

It mainly comes from the design report of the land consolidation project in Shiyan Town, Changshou District; the budget book of the land consolidation project in Shiyan Town, Changshou District; China Energy Statistics Yearbook; 2006 IPCC national greenhouse gas inventory Guide (IPCC, 2006); the budget quota of Chongqing land development and consolidation project; Changshou District Statistics Yearbook; the second national land survey data; and the relevant research literature on the carbon effect calculation of land consolidation projects.

### 3.2 Data processing

The material data of the consolidation project adopts the quantities and machine shifts in the project design report; energy consumption is obtained by conversion of the project budget quota standard. The carbon emission coefficients of different materials, energy, agricultural production inputs, and so on are modified based on relevant research results and combined with the actual situation of the study area. The carbon density parameters of soil and vegetation refer to the relevant data of similar remediation areas in the existing studies. The basic data such as crop yield are the average grain yield in the project area in the beginning year and 1 year after the completion and implementation of the agricultural land consolidation project.

## 4 Method

### 4.1 Logical framework of carbon effect accounting for agricultural land consolidation projects

**FIGURE 1:**
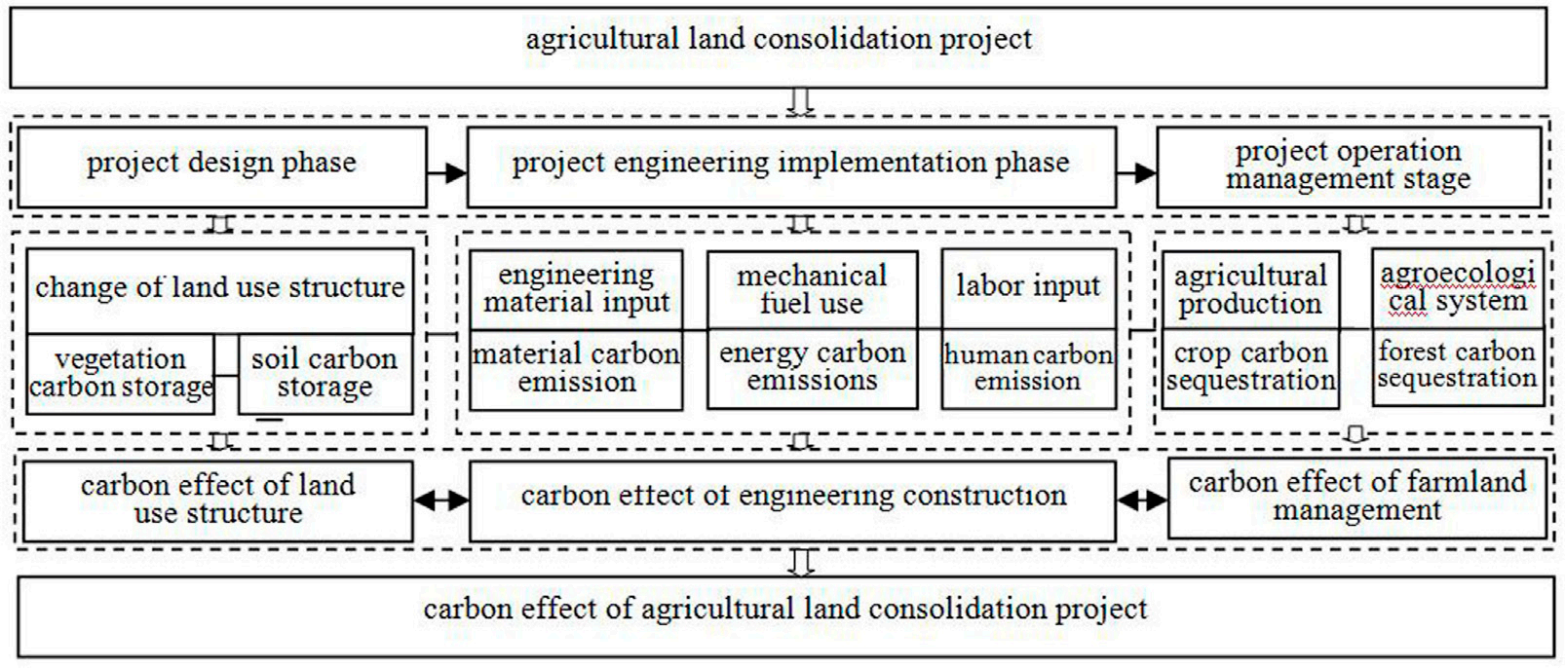
Logical framework of carbon effect accounting for agricultural land consolidation project.

Agricultural land consolidation has a profound impact on the carbon cycle and carbon storage in the project area, and the carbon effect is obvious. Based on the analysis of the whole life cycle, the agricultural land consolidation project has gone through such links as application, project approval, planning and design, engineering construction, completion acceptance, and operation. This study is simplified into three stages: project approval and design, project implementation, and operation and management. Agricultural land consolidation has an impact on biomass carbon, carbon cycle, and carbon pool reserves in the project area through disturbance of land and biomass, input of different materials, and use of mechanical fuel. The basic logic of three-stage carbon effect accounting was analyzed in the order of project life cycle (Figure 1): first, the carbon effect of land use structure change: the expected and actual land use structure change at the project approval and design stage and after completion and acceptance will lead to the change of the type and quantity of regional biomass and the disturbance of biomass carbon, resulting in the change of vegetation and soil carbon storage. Second, the carbon effect of project construction: the carbon cycle and carbon balance in the project area will be affected due to the disturbance of soil, biomass, and biomass carbon during the project construction and the disturbance of carbon balance in the project area caused by the input of a large amount of engineering materials, diesel and other fuels, and the CO_2_ emissions of personnel. The third, the carbon effect of farmland operation and management: after the completion of the project, the change of agricultural production and ecosystem operation management and protection mode, such as the change of farming activities and farmland ecosystem on the type and quantity of biomass and biomass carbon in the project area will have an impact on the soil and vegetation carbon pool.

### 4.2 Accounting method for carbon effect of agricultural land consolidation projects

#### 4.2.1 Calculation method of carbon effect in engineering construction

The engineering construction has the strongest disturbance on the soil, biomass and biomass carbon in the project area and the most direct impact on the carbon balance. One is to influence the carbon cycle in the project area by changing the land use structure and land use mode; Second, during the construction process, cement, stone, steel, concrete and other materials are put into use, diesel, electricity and other energy are consumed, and a large number of personnel are put into production, which will cause CO_2_ emissions and affect the carbon balance of the project area. The use of gasoline, diesel and other energy sources directly generates CO_2_ emissions; CO_2_ emission of cement and other materials comes from the energy consumption in the production process; CO_2_ emission of personnel input is generated from various production and construction activities. The influence of material transportation, wood and mortar plastering on carbon cycle is ignored in the carbon effect calculation, and river sand and water are partially reflected in concrete. According to this, the carbon emission during the construction of the project is the sum of the product of energy, materials, labor and its emission coefficient, the calculation model is as follows:

$$C_{eC}=\overset{n}{\underset{i=1}{\sum }}E_{i}\times f_{ie}+M_{i}\times f_{im}{+P}_{i}\times f_{ip},$$ (1)

where $C_{eC}$ is the total amount of carbon emissions from the construction of agricultural land consolidation projects; $E_{i}$ , $M_{i}$ , and $P_{i}$ are the input amount of the *i*th energy, material, and personnel during construction; $f_{ie}$ , $f_{im}$ , and $f_{ip}$ are the carbon emission coefficients corresponding to the *i*th energy, material, and personnel consumption, respectively. The existing research results (IPCC, 2006; Zhang et al., 2016; Cheng 2020; Zhai, 2017; He et al., 2018; Zhang et al., 2018) and the actual situation of the project area determine the carbon emission coefficient of each input material (Table 1).

**TABLE 1:** Carbon emission parameters of main materials/energy of agricultural land consolidation project in the project area.

Material type		Unit of measurement	Carbon emission coefficient	Data reference
Energy	Diesel oil	kg/kg	0.5927	IPCC (2006)
	Gasoline	kg/kg	0.5538	IPCC (2006); Cheng (2020)
	Electricity	kg/kw.h	0.9310	Cheng (2020)
Materials	Steel products	kg/kg	1.0600	Zhang et al. (2016); He et al. (2018)
	Cement	kg/kg	0.4598	Zhai (2017)
	Concrete	kg/m^3^	231.50	He et al. (2018)
	Block stone	kg/m^3^	2.3900	He et al. (2018)
Other	Workers	kg/人.d	18.9000	Zhang et al. (2018)
	Protection forest	kg/株.a	−23.6600	Zhang et al. (2016)

#### 4.2.2 Calculation method of carbon effect of land use structure change

The carbon effect of land use structure change is measured by the carbon storage change of the corresponding land use type before and after the consolidation. The implementation of agricultural land consolidation project will further develop and utilize some low-efficiency land such as bare land, grassland and ridge, merge small fields into large fields, level sloping farmland, build and maintain rural roads and irrigation and drainage channels, so as to adjust the land use structure and lay out of the project area, change the biomass type and production capacity, and CO_2_ emissions of the ecosystem. Referring to the research results of scholars (Zhang et al., 2016; He et al., 2018; Zhang et al., 2018), the change of carbon storage of land use types is estimated by soil carbon storage and vegetation carbon storage, that is, the change area of each land use type in the project area is multiplied by the soil carbon density and vegetation carbon density per unit area of land. The calculation model is a follows:

$${CS}_{ls}={CS}_{lb}{-CS}_{lf}=\overset{n}{\underset{i=1}{\sum }}S_{ci}\times \left({CD}_{vi},+,{CD}_{si}\right),$$ (2)

where ${CS}_{ls}$ is the total carbon storage change before and after the consolidation in the project area, ${CS}_{lb}$ and ${CS}_{lf}$ are the carbon storge after and before the consolidation, $S_{ci}$ is the area change of the *i*th land use type, ${CD}_{vi}$ and ${CD}_{si}$ are the vegetation carbon density and soil carbon density of the *i*th land use type. The determination of relevant parameters (Li et al., 2003; Chuai et al., 2012; Zhang et al., 2013; Guo et al., 2015; Cheng, 2020; Chen et al., 2021; Xiang et al., 2022) is shown in Table 2.

**TABLE 2:** Vegetation and soil carbon density parameters of different land types in the project area.

Land use type	Land type in the project area	Unit of measurement	Vegetation carbon density	Soil carbon density	Data reference
Cultivated land	Dry land and paddy field	t/hm^2^	14.30	90.8	Chuai et al. (2012); Zhang et al. (2013)
Garden plot	Orchard garden	t/hm^2^	25.10	84.3	Li et al. (2003)
Woodland	Protective forest land	t/hm^2^	23.00	113.5	Zhang et al. (2013); Li et al. (2003)
Grassland	Other grassland	t/hm^2^	10.31	109.39	Chen et al. (2021)
Land for transportation	Rural roads	t/hm^2^	2.05	33.99	Xiang et al. (2022)
Land for water and water conservancy facilities	Pond surface and channel	t/hm^2^	6.64	40.64	Cheng (2020)
Other agricultural land	Ridge of field	t/hm^2^	1.59	62.95	Guo et al. (2015)
Unused land	Bare land	t/hm^2^	1.50	55.45	Guo et al. (2015)

#### 4.2.3 Calculation method of carbon effect of project operation and management

The carbon effect of project operation and management is mainly reflected in the carbon storage of the farmland ecosystem and the carbon emission generated by the transformation of the farmland farming and management after consolidation. The carbon storage effect of farmland ecosystem lies in the increase of cultivated land area and the improvement of quality after the consolidation, which leads to the improvement of land utilization rate, crop yield, and the increase of biomass quantity and biomass carbon of crops and protective trees. The carbon sequestration capacity is calculated based on the average water content, economic coefficient, carbon absorption rate, and economic output of crops through crop biomass quantity and biochar (Zhang et al., 2016; Cheng, 2020). The calculation model of carbon absorption per unit output of crops is as follows:

$${CU}_{a}=\overset{n}{\underset{i=1}{\sum }}C_{ia}=\sum [\left(1-W_{i}\right)\times \frac{1}{H_{i}}\times f_{ia}],$$ (3)

where ${CU}_{a}$ is the carbon uptake of crops per unit output (kg/kg), $C_{ia}$ is the carbon uptake per unit crop of class i (kg/kg), $W_{i}$ and $H_{i}$ is the average water content of crops (kg/kg) and economic coefficient, and $f_{ia}$ is the carbon uptake rate (%) of class i crops. Based on the research results of scholars (Zhao and Qin 2007; Zhang Z et al., 2014; Zhang et al., 2016; Cheng, 2020; Zhang et al., 2018) and the actual situation of the project area, the relevant parameters of carbon emission are determined (Table 3).

**TABLE 3:** Carbon emission and absorption parameters of crops per unit yield in the project area.

Crops	Mean water Content/W_i_	Economic coefficient of Crops/H_i_	Carbon uptake rate of Crops/f_ia_	Carbon uptake per unit of crop Yield/C_ia_	Data reference
Paddy	0.14	0.45	0.41	0.783	Zhao and Qin, (2007); Zhang et al. (2018)
Corn	0.13	0.40	0.47	1.065	Cheng (2020); Zhang et al. (2018)
Wheat	0.13	0.40	0.49	1.066	Zhang et al. (2016); Zhang et al. (2018)
Rapeseed	0.09	0.25	0.45	1.638	Cheng (2020); Zhang T et al. (2014)

During the operation and management of the project, various agricultural production activities will inevitably produce CO_2_ emissions. First, the carbon emissions from agricultural production links, such as the CO_2_ emissions from energy consumption of farming machinery, crop irrigation and drainage, and the use of fertilizers and pesticides. The second is the emission of greenhouse gases from farmland ecosystems, such as soil respiration and CH_4_ emissions from paddy fields. In practice, material balance algorithm and farmland greenhouse gas flux are usually used to estimate (Cheng (2020); Song, 2017). The calculation formula is as follows:

$$CE={\overset{n}{\underset{i=1}{\sum }}{CE}_{i}=\overset{n}{\underset{i=1}{\sum }}E}_{i}\times \gamma_{i},$$ (4)

where CE is the total amount of carbon emissions from agricultural cultivation in the project area, ${CE}_{i}$ is the carbon emission of agricultural farming activities or agricultural inputs of type i , $E_{i}$ is the amount of agricultural activities or agricultural inputs of type i , and $\gamma_{i}$ is the carbon emission coefficient of different agricultural activities. The corresponding carbon emission coefficient is determined according to the research of scholars (Zhang T et al., 2014; Zhao and Qin (2007); Song, 2017; Duan et al., 2011; Wu et al., 2007) and the actual situation of the project area (Table 4).

**TABLE 4:** Main carbon emission parameters of agricultural production activities in the project area.

Agricultural production activities	Carbon emission coefficient	Unit of measurement	Data reference
Agricultural irrigation	266.48	kg/hm^2^	Duan et al. (2011); Zhao and Qin, (2007)
Land cultivation	218.82	kg/km^2^	Zhang et al. (2018); Wu et al. (2007)
Fertilizer application	0.8956	kg/kg	Zhang T et al. (2014); Zhang et al. (2018)
Pesticide spraying	4.9341	kg/kg	Zhang Z et al. (2014); Zhang et al. (2018)
Agricultural film mulching	5.18	kg/kg	Zhang Z et al. (2014); Zhang et al. (2018)

## 5 Results and analysis

### 5.1 Carbon effect of project construction

**FIGURE 2:**
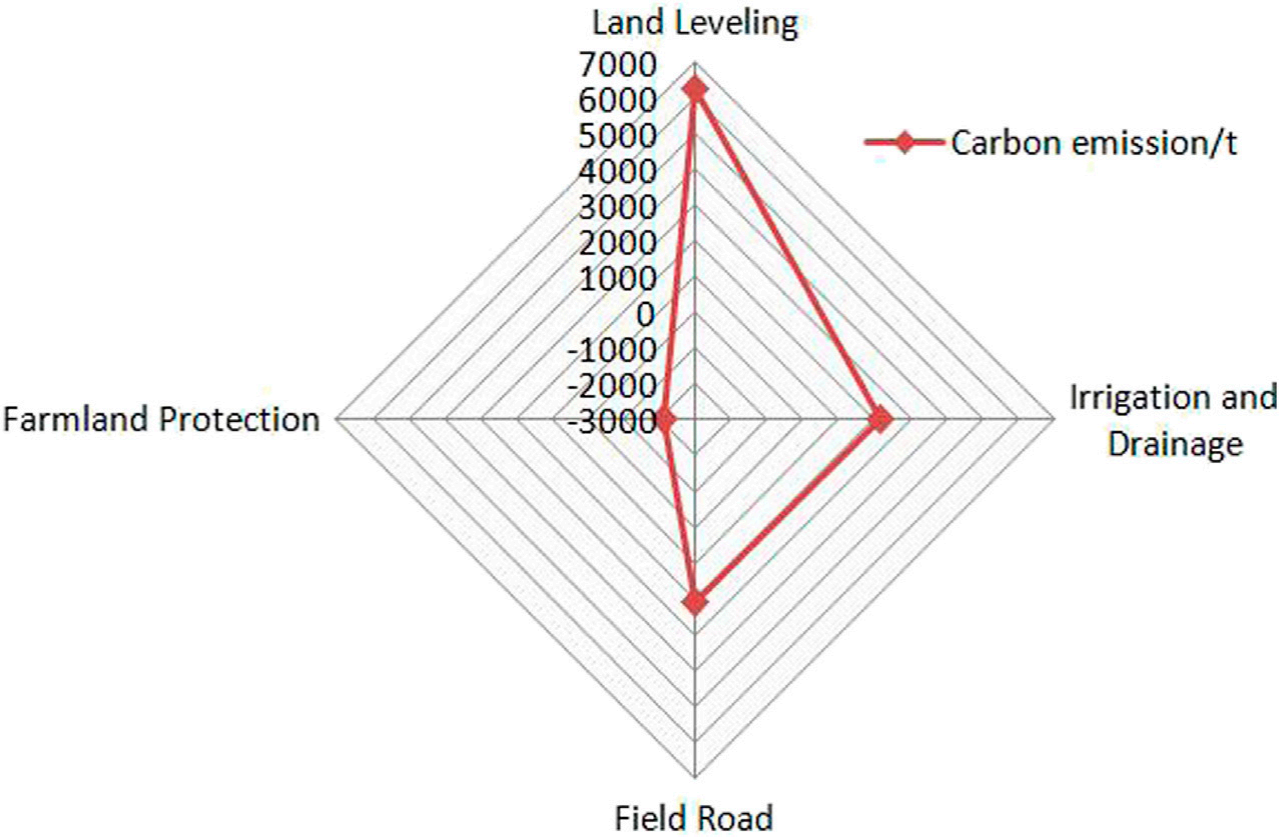
Carbon effect structure of different land consolidation projects (including personnel).

**FIGURE 3:**
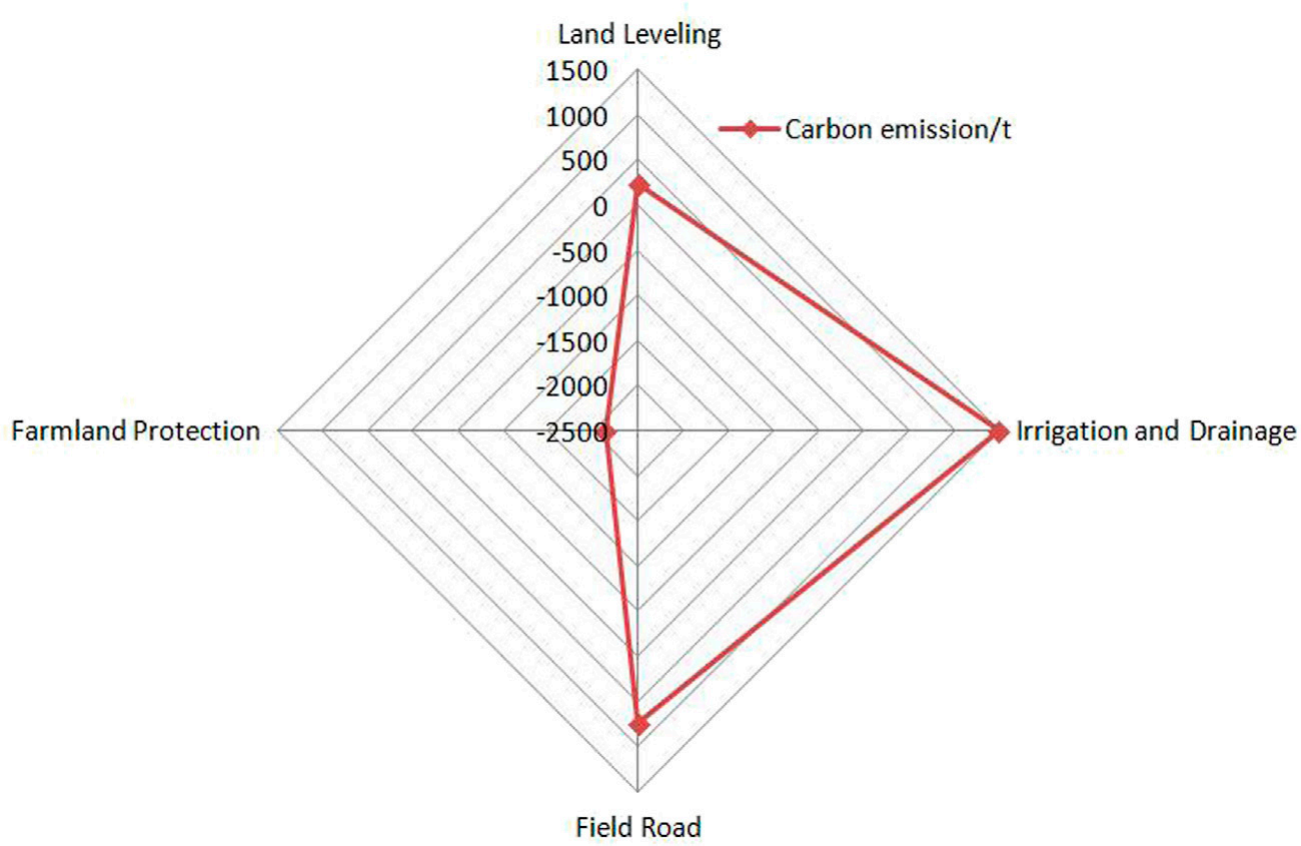
Carbon effect structure of different remediation projects (excluding personnel).

The calculation results showed that the carbon emission of the project construction was 10,493.5t, and the carbon sink is 2135t, On the whole, it was shown as carbon source, and the net carbon emission was 8358t (Table 5). The emission of energy was 72.06t, accounting for 0.69% of the total carbon emission, gasoline 4.82t, diesel 19.27t, and electricity 47.97t, accounting for relatively low. The emission of materials was 2,410.87t, accounting for 22.97%, steel 25.20t, cement 344.98t, concrete 1,598.8t, block stone 441.89t, concrete was the most. Other categories: first, artificial emission of 8010.6T, accounting for 76.34% of the total, which is the main body of carbon emission. The second is the shelter forest project, which stores 2135t carbon, which can offset 20.35% of the total carbon emission. In terms of project type, the carbon emission from land leveling is 6249t, accounting for 59.55% of the total carbon emission; Irrigation and water conservancy 2170t, accounting for 20.68%; and field road 2074t, accounting for 19.77%. Farmland protection works are carbon sinks (Figure 2). If the impact of artificial carbon emission is not included, the total carbon emission is 2,481.97t, including 1,491.36t for farmland water conservancy projects, 749.23t for field road projects and 241.38t for land leveling projects (Figure 3).

**TABLE 5:** Construction materials, energy input, and carbon emission results of the project area.

Material type		Land leveling		Irrigation and drainage		Field road		Farmland protection		Total	
		Consumption	Carbon emission	Consumption	Carbon emission	Consumption	Carbon emission	Consumption	Carbon emission	Consumption	Carbon emission
Energy	Diesel oil	0.40	0.24	9.86	5.84	22.26	13.19	0.00	0.00	32.52	19.27
	Gasoline	0.00	0.00	7.31	4.05	1.39	0.77	0.00	0.00	8.70	4.82
	Electricity	0.00	0.00	26.47	24.64	25.05	23.32	0.00	0.00	51.52	47.97
Materials	Steel products	0.00	0.00	23.77	25.20	0.00	0.00	0.00	0.00	23.77	25.20
	Cement	0.00	0.00	495.0	227.6	255.3	117.4	0.00	0.00	750.3	344.98
	Concrete	0.00	0.00	4,974	1,151	1928	446	0.00	0.00	6,902	1,598.8
	Block stone	100.9	241	22.01	52.61	61.99	148.1	0.00	0.00	184.9	441.89
Other	Worker	31.78	6,007	3.59	678.9	7.01	1,325	0.00	0.00	42.38	8010.6
	Protection forest	0.00	0.00	0.00	0.00	0.00	0.00	90,230	−2,135	90,230	−2,135
小计			6,249		2,170		2074		−2,135		8358

Unit of measurement: Electricity 1000Kw.h, concrete block stone 1000m^3^, worker/10000, shelterbelt/plant, steel products, diesel oil, gasoline, cement, carbon emission/t.

The project area is located in the hilly area on the edge of the metropolitan area of the Three Gorges Reservoir area. The project construction involves a lot of labor, with a total of 423,800 people per day, and less machinery. Therefore, different from many existing scholars, this study fully considered artificial carbon emission during construction. At the same time, the farmland protection project will be included in the carbon effect accounting of the project construction stage, and the carbon sink function will be played through the accumulation of biomass quantity and biomass carbon.

### 5.2 Carbon effect of land use structure change

After renovation, the cultivated land in the project area has increased by 54.15 h m^2^, including 37.05 h m^2^ of dry land, 2.36 h m^2^ of paddy field, 2.36 h m^2^ of garden land, 4.28 h m^2^ of rural road land, the water surface of pit pond remains unchanged, 1.36 h m^2^ of ditch, 0.48 h m^2^ of other grassland, and 36.94 h m^2^ and 25.69 h m^2^ of ridge and bare land (Table 6). Rural residential areas, roads and woodlands are not included in the project area.

**TABLE 6:** Carbon storage change for land use structure adjustment in the project area.

Land type		Before consolidation	After consolidation	Land type change	Carbon storage change		
					Soil	Vegetation	Subtotal
Cultivated land	Dry land	180.32	217.37	37.05	3,364.14	529.82	3,893.96
	Paddy field	177.61	194.71	17.10	1,552.68	244.53	1797.21
Garden plot	Orchard garden	0.00	2.36	2.36	198.95	59.24	258.19
Land for transportation	Rural roads	5.29	9.57	4.28	145.48	8.77	154.25
Land for water and water conservancy facilities	Pond surface	1.72	1.72	0.00	0.00	0.00	0.00
	Channel	0.51	1.87	1.36	55.27	9.03	64.30
Grassland	Other grassland	0.00	0.48	0.48	52.51	4.95	57.46
Other agricultural land	Ridge of field	162.69	125.75	−36.94	−2,325	−58.73	−2,384
Unused land	Bare land	25.69	0.00	−25.69	−1,424	−38.53	−1,463
Total		553.83	553.83	0.00	1,619.14	−59.06	2,378.20

Unit of measurement: land type/hm^2^, soil, and vegetation carbon storage/t.

The calculation results showed that the carbon storage increment generated by the change of land use structure was 2,378.20t, which was a carbon sink in general. On the change of carbon storage of different land use types, the carbon storage of cultivated land increased by 5,691.17 t (3,893.96 t for dry land and 1797.21 t for paddy field), 258.19 t for garden land, 154.25 t for rural roads, 64.30 t for irrigation and drainage ditches, 57.46 t for grassland. The carbon storage of the ridge of field is reduced by 2384t, and the unused land is reduced by 1463t (Table 6). Increase of carbon reserves in the project area is mainly due to the increase of land use types such as dry land, paddy field, garden land and rural roads due to the improvement of other agricultural land and the development of unused land, thus increasing the biomass production, soil organic matter and biomass charcoal in the project area.

### 5.3 Carbon effect of project operation and management

#### 5.3.1 Carbon sink effect of farmland ecosystem

The main grain crops in the project area are paddy, wheat and corn, and the major cash crops are rapeseed and some vegetables. In agricultural production, the rotation of corn and wheat is adopted in dry land, and paddy and rapeseed are rotated in paddy field. After the implementation of the project, firstly, the increase of the amount of arable land leads to the increase of grain output; secondly, the improvement of the medium and low yield fields leads to the improvement of the quality of arable land and the increase of crop output. Both of them work together to increase the overall biomass, biomass carbon and carbon reserves in the project area due to the increase of grain output. According to the calculation results, the newly increased cultivated land and grain yield: paddy field 17.1 h m^2^, dry land 37.5 h m^2^, rice 128.76t, rapeseed 38.48t, corn 161.17t, wheat 84.47t, a total of 412.88t; Arranging medium and low yield fields and increasing grain yield: arranging 180.32 h m^2^ of dry land and 177.76 h m^2^ of paddy fields, increasing the yield of rice by 213.3t, rapeseed by 259.41t, corn by 143.76t and wheat by 148.76t, totaling 625.13t (Table 7).

**TABLE 7:** Change of crop yield and carbon storage after consolidation in the project area.

Crops	Grain output of newly increased cultivated land		Increase grain yield by arranging medium and low yield fields		Carbon storage
	Cultivated land area	Grain yield	Renovation area	Grain production increase	
Paddy	17.1	128.76	177.61	213.13	267.70
Rapeseed		38.48		119.89	259.41
Corn	37.05	161.17	180.32	143.35	324.31
Wheat		84.47		148.76	248.62
合计	54.15	412.88	357.93	625.13	1,100.04

Unit of measurement: area/hm^2^, grain yield, grain production increase, and carbon storage/t.

The results showed that the increase of crop biomass production in the project area increased the total carbon storage by 1,100.04t, including 267.70t of rice, 259.41t of rapeseed, 324.31t of corn and 248.62t of wheat. In general, the proportion of carbon storage increase caused by the consolidation of medium and low yield fields in the project area was larger (Table 7), and the carbon sink effect of agricultural ecosystem was obvious.

#### 5.3.2 Carbon source effect of agricultural production activities

After the implementation of the consolidation in the project area, the amount of fertilizer, pesticide, agricultural film and other materials invested in agricultural farming activities is adjusted every year, and the area of agricultural irrigation and land cultivation is increased, resulting in the change of carbon balance in the project area. The results showed that the agricultural irrigation area increased by 46.33 h m^2^, the land plowing increased by 0.57 km^2^, the use of agricultural film increased by 4.49t, and the carbon emission increased by 12.35 t, 0.12 t and 23.26 t respectively in 1 year; The amount of pesticide application was reduced by 0.55t and the amount of fertilizer was reduced by 16.56t. The annual carbon emissions in the project area are reduced by 2.71t and 14.83 T respectively. In general, the annual net carbon emission of agricultural production activities in the project area after consolidation was 18.18t, which was shown as carbon source effect (Table 8).

**TABLE 8:** Agricultural production activities and carbon emissions after consolidation in the project area.

Agricultural production activities	Unit of measurement	Before consolidation	After consolidation	Quantity change	Carbon Emissions/t.a
Agricultural irrigation	hm^2^	221.63	267.96	46.33	12.35
Land cultivation	km^2^	3.57	4.14	0.57	0.12
Fertilizer application	t	268.45	251.89	−16.56	−14.83
Pesticide spraying	t	3.98	3.43	−0.55	−2.71
Agricultural film mulching	t	20.4	24.89	4.49	23.26
Total		518.03	552.31	34.28	18.18

### 5.4 Carbon effect balance analysis in the project area

The carbon effect of the three stages of the agricultural land consolidation project is different. During the implementation stage of the agricultural land improvement project, due to various materials input, machinery use, energy consumption, labor input and other reasons, the ecosystem, biomass production and biomass carbon in the project area are destroyed, resulting in a large amount of carbon emissions, affecting the regional carbon cycle and carbon balance. The carbon emission in this stage is short-term and one-time, and the project area is a carbon source; In the stage of operation management and conservation, due to the increase of cultivated land area, quality improvement, biomass production of crops and biomass carbon, it is manifested as a carbon sink, which is a long-term carbon effect with an annual cycle. At the same time, in the stage of operation and management, agricultural production activities increased, and the overall carbon emissions increased accordingly. In this cycle, after a certain period of operation, the carbon emissions from the project construction will be gradually digested by the agricultural ecosystem to achieve carbon balance. The time to achieve carbon balance is determined by the carbon emission of project construction, the carbon storage of land use structure adjustment, the carbon storage of agroecosystem and the carbon emission of agricultural production activities. The calculation formula is as follows:

$$T_{cb}=\frac{C_{eC}-{CS}_{ls}}{{CU}_{a}-CE},$$ (5)

where $T_{cb}$ is the time to achieve carbon effect balance in the project area, $C_{eC}$ is the total amount of carbon emissions from the construction of agricultural land consolidation project, ${CS}_{ls}$ is the total carbon storage change before and after the consolidation in the project area, ${CU}_{a}$ is the carbon uptake of crops per unit output, and CE is the total amount of carbon emissions from agricultural cultivation in the project area.

The carbon emissions of the project construction are 8358t, the carbon reserves of land use structure adjustment are 2,378.20t, the carbon reserves of agricultural ecosystem in the project area are 1,100.04t, and the carbon emissions of agricultural production activities are 18.18t. It is estimated that the time to achieve carbon balance is 5.53 years.

## 6 Conclusion and discussion

### 6.1 Conclusion

Based on the historical background of promoting the construction of ecological civilization and achieving the goal of carbon peak and carbon neutralization, this study took the life cycle of agricultural land consolidation project as the clue, and adopted quantitative analysis method to construct the carbon effect accounting and analysis framework of agricultural land consolidation project from three stages of project approval design, project implementation and operation and management. Taking the agricultural land consolidation project in Shiyan town on the edge of the Three Gorges Reservoir Area as the research case, this study made empirical analysis and calculation, and explored the influencing factors of carbon storage in different life cycle stages. The main conclusions were as follows:

(1) The overall carbon effect of the project area was measured as the carbon source state. The net carbon emissions generated by the project construction were 8358t, the carbon reserved generated by the land use structure adjustment were 2,378.20t, the carbon reserved generated by the agricultural ecosystem were 1,100.04t, and the carbon emissions from agricultural production activities were 18.18t.
(2) The project area was located in the hilly area on the edge of the metropolitan area of the Three Gorges Reservoir area, and the carbon source effect of the project construction was obvious. Among them, the carbon emission of artificial input was 8010.6t, and the land leveling project in the project category was 6249t, accounting for 59.55% of the total carbon emission; excluding the carbon emission of artificial input, the total carbon emission was 2,481.97t, of which 1,598.8t was from concrete, accounting for 64.42%, followed by 441.89t from block stone and 344.98t from cement. In the project category, 1491.36t was from farmland water conservancy, 749.23t was from field roads and 241.38t was from land leveling. The farmland shelter forests were carbon sinks, increasing carbon reserves by 2135t.
(3) The carbon effect of land use structure adjustment was generally shown as carbon sink. Among them, the newly increased cultivated land in the project area increases the carbon reserved by 5,691.17t, which was the main body of the increase in carbon reserves; As the area of ridge of field and bare land decreases, the carbon reserves were reduced by 2384t and 1463t.
(4) The calculation results of the carbon protection effect of the project operation and management were generally shown as carbon sinks. The carbon reserved of farmland ecosystem increased by 1,100.04t due to the increase of cultivated land area and the improvement of cultivated land quality, and the carbon reserved increased more due to the improvement of cultivated land quality. The total carbon emission from agricultural production activities was 18.18t.

### 6.2 Discussion and suggestions

Under the background of vigorously promoting the construction of ecological civilization, rural land consolidation will pay more and more attention to the goal of low carbon emission and high carbon sink. Rural land consolidation will certainly have a certain reverse effect on ecosystem structure and carbon sequestration effect in the project area. Therefore, it is inevitable to explore and promote low carbon and ecological land consolidation.

(1) Ecological land consolidation is an important means to promote the construction of ecological civilization and the realization of the “double carbon” goal. The research shows that the different stages of agricultural land consolidation, especially the engineering construction, have strong disturbance on the soil and vegetation in the project area, and great influence on the biomass production of the ecosystem, which directly affects the regional carbon cycle and carbon balance. Therefore, exploring and promoting ecological land consolidation with low carbon emission and high carbon sink is an important carrier to achieve the “double carbon” goal and an important means to promote the construction of ecological civilization.
(2) Based on the concept of life cycle, optimizing project design, reasonably arranging consolidation projects and strengthening operation management and protection are effective measures to improve the carbon effect of land consolidation projects.

Project approval and design stage: revise the policy standards for land consolidation project approval, and take the potential of carbon sequestration and sink increase as an important indicator for project approval and storage. In the operation, the change of carbon storage in vegetation and soil and carbon effect accounting of the project area before and after the consolidation are taken as the necessary contents of the project feasibility study, and the projects with good carbon effect and strong implement ability are preferentially selected for storage and filing.

Construction stage of the project: ecological engineering materials such as ecological bricks are mostly selected. The layout of the project is adapted to local conditions to reduce unnecessary or small projects. The projects that are unreasonable or have a great impact on the ecological environment of the project area are canceled according to the actual situation. The terrain and landform of the project area are mostly kept, and the demolition and construction are not large, so as to reduce the engineering disturbance to the project area.

Project operation and management stage: establish and adjust the acceptance and assessment standards for agricultural land consolidation projects, and take the achievement degree and stability of carbon reduction and sink increase of newly added cultivated land, farmland water conservancy projects, field road projects and farmland shelter forest projects before and after the implementation of the project as important indicators. Regional standards and incentives for farmland cultivation, seed selection, irrigation and drainage, fertilizer and pesticide application should be established to reduce carbon emission and increase carbon storage, so as to improve the carbon storage and carbon effect of the project area through a perfect operation management and conservation policy system.

## Data Availability

The original contributions presented in the study are included in the article/Supplementary Material; further inquiries can be directed to the corresponding authors.
